# 

**Published:** 1882-11

**Authors:** 


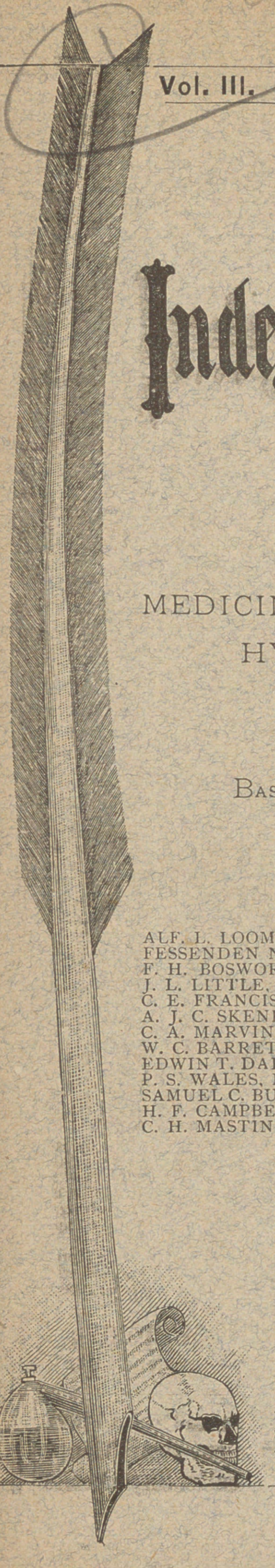

Vol. III. NOVEMBER, 1882. No. 11.

THE

### Independent Practitioner:

A MONTHLY JOURNAL,

DEVOTED TO

MEDICINE, SURGERY, OBSTETRICS, DENTISTRY, HYGIENE, AND POPULAR SCIENCE.

S.G.O. INDEXED

EDITORIAL CORPS:

BASIL M. WILKERSON, D.D.S., M.D., EDITOR.

Associates:

——— M.D. ——— D.D.S.

NEW YORK:

*THE INDEPENDENT PRACTITIONER CO.,*

VANDERBILT BUILDING,

132 NASSAU STREET.

**$3.00 Per Annum, in Advance. - - - Single Copies, 30 Cents.**

*Entered at the Post-Office, New York City, as Second-Class matter.*

